# Demulsification of Fluids Produced from Polymer Flooding in Oilfields: A Molecular Dynamics Simulation Study

**DOI:** 10.3390/ma19061181

**Published:** 2026-03-17

**Authors:** Qian Huang, Zhe Shen, Yuxin Xie, Lingyan Mu, Xueyuan Long, Jiang Meng, Xicheng Zhang, Ruilin Wang

**Affiliations:** 1 School of Petroleum Engineering, Chongqing University of Science and Technology, Chongqing 401331, China; huangqianswpu@163.com (Q.H.); xieyuxincqust@163.com (Y.X.); cqmj07@163.com (J.M.); tangchengcai91@gmail.com (X.Z.); 2024201088@cqust.edu.cn (R.W.); 2 Nan’an Branch, Chongqing Gas Group Co., Ltd., Chongqing 400060, China; 13653895177@163.com; 3 School of Safety Science and Engineering, Chongqing University of Science and Technology, Chongqing 401331, China

**Keywords:** molecular dynamics simulation, polymer flooding, interfacial behavior, polyether demulsifier

## Abstract

In this study, a combined approach of molecular dynamics (MD) simulations and experimental bottle tests was employed to systematically investigate the demulsification performance and underlying mechanisms of two distinct demulsifiers—Demulsifier X (SP/BP series and alcohol-initiated polyethers) and Demulsifier Y (AP/AE series and amine-initiated polyethers)—targeting polymer-containing oil-in-water (O/W) emulsions derived from heavy oil polymer flooding. Molecular models for heavy oil, saline water, partially hydrolyzed polyacrylamide (HPAM), and demulsifiers were constructed using BIOVIA Materials Studio software. Their dynamic behaviors at the oil–water interface were simulated within three distinct saline systems containing NaCl, CaCl_2_, and MgCl_2_, respectively. Simulation results indicated that the demulsifiers effectively displaced interfacial HPAM molecules, increased interfacial tension, and reduced interfacial interaction energy. Experimental bottle tests, evaluating the effects of settling time, temperature, and concentration on dehydration rates and oil content, confirmed that Demulsifier Y outperformed Demulsifier X. Specifically, Demulsifier Y achieved superior dehydration rates with lower dosages, shorter settling times, and reduced temperature requirements under optimal conditions. This work provides both microscopic mechanistic insights and macroscopic experimental validation for the screening and application of high-efficiency demulsifiers.

## 1. Introduction

Heavy oil constitutes a cornerstone of China’s stable petroleum supply, accounting for approximately 8% of the national annual oil output. Although polymer flooding has been widely applied to enhance heavy oil recovery, it inevitably generates large quantities of polymer-containing oil-in-water (O/W) emulsions with high stability, which severely restricts efficient oil–water separation. Residual partially hydrolyzed polyacrylamide (HPAM) reinforces the viscoelastic interfacial film, suppresses droplet coalescence, and significantly impairs downstream surface processing. Therefore, elucidating the microscopic stabilization mechanisms of polymer-containing emulsions and developing efficient demulsification strategies are of great practical importance.

The fundamental objective of demulsification is to disrupt the stabilization mechanisms of the emulsion. Core strategies focus on modulating the evolution of oil–water interfacial film properties and understanding the structure–activity relationship (SAR) between demulsifier molecules and phase characteristics. Although physical and biological methods exist [1], chemical demulsification remains the dominant industrial approach due to its superior efficiency. Chemical demulsifiers, categorized into ionic, non-ionic, and composite types [2], typically drive kinetics through four stages: flocculation, drainage, film rupture, and coalescence [3]. From a materials science perspective, current academic discourse centers on how these molecules alter interfacial rheology [4]. However, due to the complexity of crude oil components and demulsifier structures, a unified mechanistic model linking molecular architecture to macroscopic demulsification performance has yet to be established [5]. Historically, research on crude oil emulsion stability has relied heavily on macroscopic experimental techniques, such as interfacial tension and rheology measurements. While these approaches have established classical stability theories, they struggle to directly resolve dynamic interactions at the atomic scale, hindering the revelation of the microscopic origins of interfacial behaviors [6,7,8,9,10,11]. To address this, Molecular Dynamics (MD) simulation has emerged as a critical bridge spanning the gap between macroscopic phenomena and microscopic mechanisms [12].

Since the 1970s, MD simulations have matured in characterizing complex fluids. By constructing precise molecular force fields, this method enables the quantitative analysis of adsorption, diffusion, and aggregation behaviors at the oil–water interface [13]. Recent MD studies have provided significant insights into asphaltene aggregation and demulsifier mechanisms. For instance, Li et al. [14] and Wang [15] investigated the solvent effects on asphaltene kinetics and the “peeling” mechanism of ethyl cellulose, respectively. Similarly, Niu et al. [16] and Santos et al. [17] revealed the competitive adsorption and hydration mechanisms of various demulsifiers. Geng et al. [18] further highlighted the structural stability of fluorinated polyethers. Recently, an increasing number of studies have utilized MD simulations to design and screen novel demulsifiers. For example, recent works have demonstrated how specific molecular interactions, such as hydrogen bond reconstruction and the introduction of novel demulsifier architectures, dictate interfacial penetration kinetics and effectively disrupt rigid emulsion films [19,20]. Furthermore, cutting-edge simulations coupled with experimental validations have deeply explored the dynamic competitive replacement of natural or polymer surfactants by chemical and biological demulsifiers under complex conditions [21,22].

Despite these advances, a specific knowledge gap remains: MD simulation studies specifically targeting the complex multicomponent system of fluids produced by polymer flooding (containing HPAM, crude oil, and water) are scarce. The specific molecular mechanisms by which residual HPAM interacts with demulsifiers to influence interfacial film stability have not been systematically clarified. Therefore, this study employs molecular dynamics simulations to investigate the interfacial behavior of HPAM-stabilized O/W emulsions. By analyzing molecular adsorption characteristics, structural evolution, and interaction energies, the primary contribution of this work lies in elucidating the microscopic competitive adsorption mechanisms between residual HPAM and polyether demulsifiers. By bridging atomic-scale interaction energies with macroscopic dehydration rates, this study provides direct theoretical guidance for the rational screening and design of high-efficiency demulsifiers in industrial polymer-flooding applications.

## 2. Materials and Methods

### 2.1. Molecular Models of the Base Components

Molecular dynamics (MD) simulations were conducted using BIOVIA Materials Studio 2024 (Dassault Systèmes, San Diego, CA, USA) to systematically examine emulsion stability in fluids produced by polymer flooding and elucidate the underlying demulsification mechanisms. Molecular models for the key components—heavy oil, saline water, polymers, and ions—were constructed following the protocols and parameter settings established in our previous work [23]. The accuracy of these models has been rigorously validated against macroscopic properties such as density, with relative errors below 1.5%. The key specifications of these established models are summarized in Table 1.

### 2.2. Demulsifier Molecular Models

Building upon previously validated models, this study constructs representative molecular models of demulsifiers to elucidate their microscopic mechanisms at the oil–water interface. Given the high concentration of asphaltenes and resins in the target heavy oil, two industrially prominent categories were selected: Type X (SP/BP series) and Type Y (AP/AE series), representing alcohol-initiated and amine-initiated polyether demulsifiers, respectively.

The SP/BP series typically comprises linear block polyethers synthesized from fatty alcohols or alkylphenols, known for their favorable wettability. In contrast, the AP/AE series (often referred to as AP/EP in industrial contexts) features highly branched, multi-arm architectures that provide superior interfacial activity and more rapid penetration into rigid viscoelastic films. To bridge the molecular models with macroscopic behavior, the key physicochemical parameters of these demulsifiers—including molecular weight, HLB, solubility, and interfacial tension—are summarized in Table 2.

#### 2.2.1. Alcohol-Initiated Polyether Demulsifiers (SP and BP Series)

**Figure 1:**
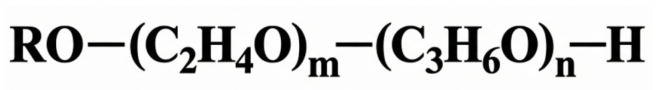
Structural formula of SP and BP series demulsifiers.

Based on the established literature [25], a representative alcohol-initiated demulsifier widely used in oil field applications was selected for this study. These demulsifiers are synthesized through the sequential polymerization of propylene oxide (PO) and ethylene oxide (EO) using alcohols as initiators. The general chemical formula is R-(PO)m-(EO)n-H, where m and n represent the degrees of polymerization. The specific molecular structure is illustrated in Figure 1.

#### 2.2.2. Amine-Initiated Polyether Demulsifiers (AP and AE Series)

**Figure 2:**
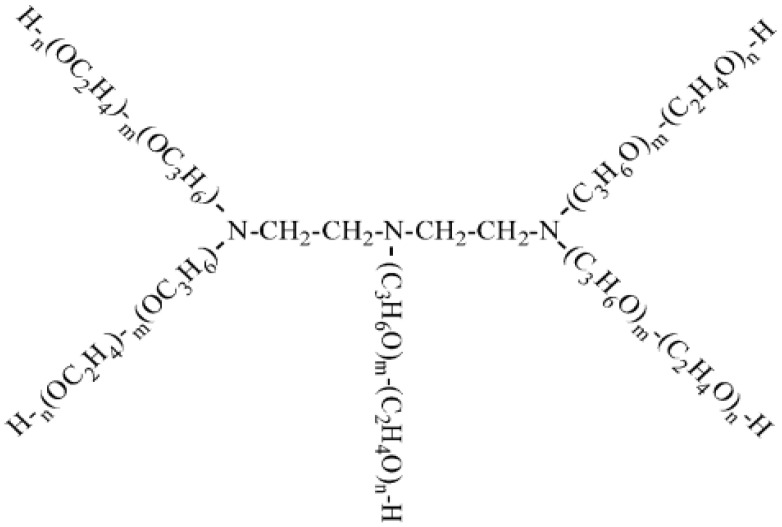
Structural formulas of AP and AE series demulsifiers.

Amine-initiated demulsifiers are similarly synthesized via the sequential polymerization of EO and PO but utilize amines as initiators. This class typically outperforms conventional alcohol-based counterparts, demonstrating significant effectiveness in treating high-wax emulsions [26]. A representative chemical structure is presented in Figure 2.

**Figure 3:**
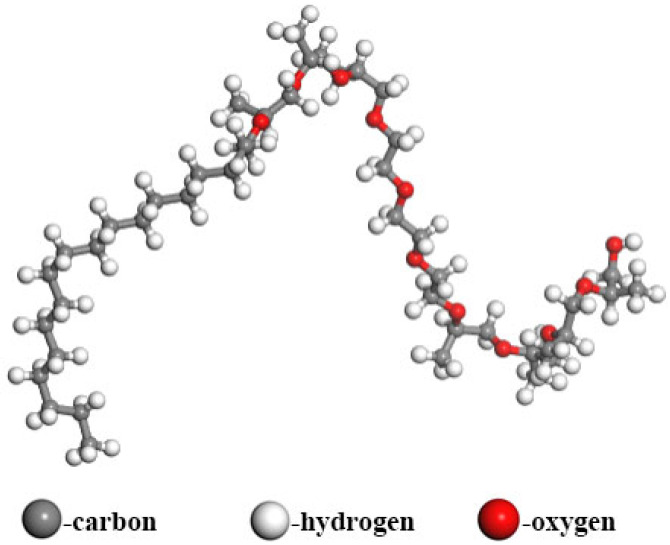
Molecular model of X demulsifier.

**Figure 4:**
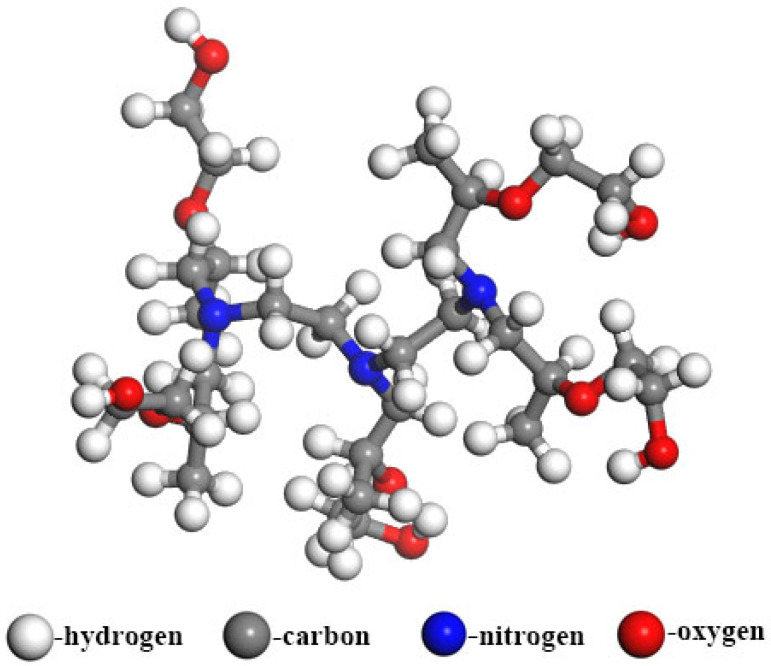
Molecular model of Y demulsifier.

Following the structural characteristics described above, 3D molecular models for both the SP-type and AE-type demulsifiers were constructed using BIOVIA Materials Studio software. Because the specific degrees of polymerization (m and n) critically dictate demulsifier properties, the molecules in this study were built with a 1:1 molar ratio of EO to PO units along the polymer chain to ensure comparability. The resulting atomic models, designated as Demulsifier X (alcohol-initiated) and Demulsifier Y (amine-initiated), are illustrated in Figure 3 and Figure 4, respectively.

### 2.3. Molecular Dynamics Simulation Methods

This study employed Materials Studio software and a molecular dynamics (MD) simulation approach to systematically investigate the stability of emulsions in fluids produced from polymer flooding and the microscopic mechanisms of demulsifiers. The overall simulation followed a standardized workflow comprising structure modeling, system construction, energy minimization, equilibration, and property analysis.

Specifically, all-atom molecular models of each component, including heavy oil, saline water, polymers, and demulsifiers, were first constructed. The composite simulation systems were then assembled using the Amorphous Cell module with periodic boundary conditions. Geometry optimization and annealing were subsequently performed based on the COMPASS III force field to minimize system energy and eliminate unfavorable atomic contacts. Afterward, MD simulations were carried out under NVT and NPT ensembles. For readers less familiar with MD simulation terminology, the NVT ensemble (isothermal–isochoric process) maintains a constant Number of particles, Volume, and Temperature, allowing the mixed system to initially relax its molecular conformations without boundary volume changes. Subsequently, the NPT ensemble (isothermal–isobaric process) maintains a constant Number of particles, Pressure, and Temperature. This step allows the simulation box volume to adjust dynamically, accurately reflecting real-world macroscopic pressure conditions (e.g., standard atmospheric pressure). The simulations were run continuously under these conditions until complete thermodynamic equilibrium was achieved. Finally, equilibrium trajectories were analyzed to evaluate interfacial properties, interaction energies, and other microscopic indicators relevant to demulsification behavior.

### 2.4. Experimental Validation Methods

Crude oil and water samples were obtained from the S Oilfield. The aqueous phase was prepared by dissolving HPAM (200 mg/L) into a synthetic brine solution. To prepare the emulsion, 20 g of crude oil was mixed with 180 g of the aqueous phase. The mixture was stirred in a beaker with a liquid volume of 350 mL, with the stirring impeller positioned 10 mm above the bottom. The emulsification process was conducted at 1000 rpm for 15 min at 60 °C, resulting in a stable oil-in-water (O/W) heavy oil emulsion.

The demulsification performance was evaluated using the bottle test method in accordance with the industrial standard [27]. The prepared O/W emulsion (100 mL) was transferred into stoppered graduated cylinders. Demulsifiers were added at various concentrations, and the cylinders were subjected to 100 horizontal shakes using an oscillator. Subsequently, the cylinders were placed in a constant-temperature environment for static settling. The dehydration rate was calculated based on the volume of separated water, and the oil content in the aqueous phase was determined using infrared spectrophotometry.

## 3. Results and Discussion

### 3.1. Molecular Dynamics Simulation of the Demulsification Process

Based on the molecular models and simulation protocols established in Section 2, molecular dynamics (MD) simulations were performed to evaluate the effects of two representative demulsifiers—Demulsifier X (SP/BP series) and Demulsifier Y (AP/AE series)—on the interfacial properties of polymer-containing oil–water emulsions. Furthermore, the influence of various inorganic brine environments (NaCl, CaCl_2_, and MgCl_2_) was systematically investigated. For clarity and conciseness, the calculated interfacial tensions and average interfacial interaction energies across all simulated systems are summarized in Table 3 and Table 4, respectively.

#### 3.1.1. Interfacial Behavior of Demulsifier X Systems

**Figure 5:**
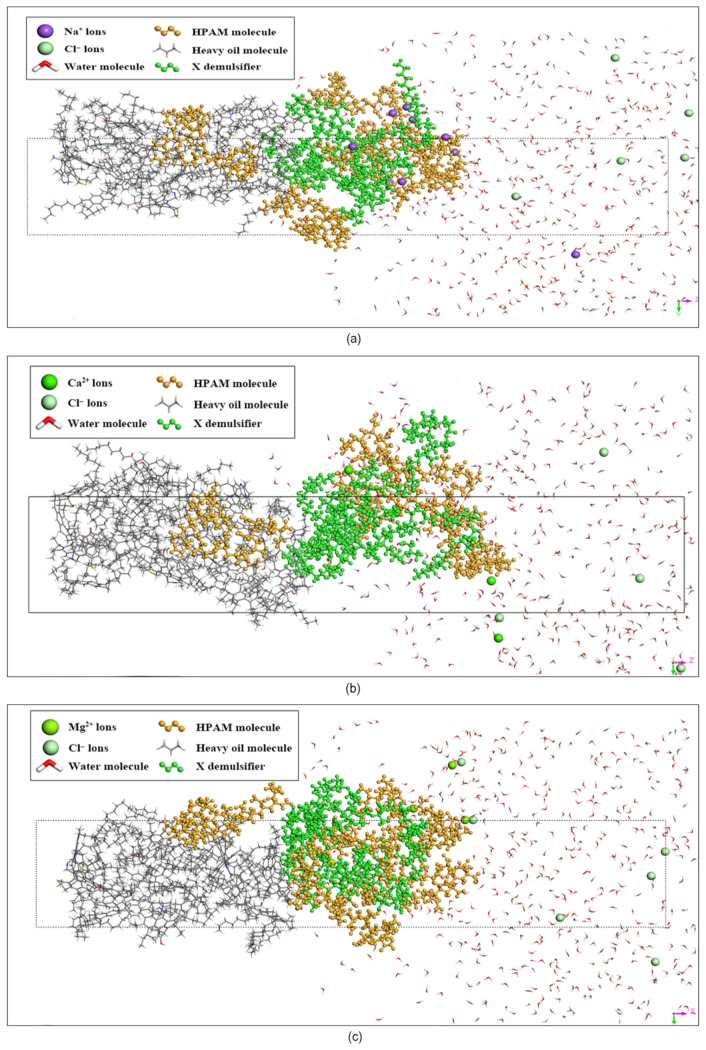
Equilibrium snapshots of the molecular dynamics simulation systems: (**a**) X-NaCl; (**b**) X-CaCl_2_; (**c**) X-MgCl_2_. The molecular components are distinguished by color: Demulsifier X (green), HPAM chains (orange), heavy oil (grey), water (red/white), and respective ions (spheres). The dotted box indicates the boundaries of the simulation cell.

Figure 5a, Figure 5b and Figure 5c present the equilibrium snapshots of the simulation systems containing Demulsifier X in the NaCl, CaCl_2_, and MgCl_2_ brine environments, respectively. Driven by their inherent amphiphilic nature and superior interfacial activity, the Demulsifier X molecules (green chains) spontaneously migrated to and effectively penetrated the phase boundary. As the systems reached thermodynamic equilibrium, these demulsifier molecules dominated the oil–water interface, competitively displacing the originally adsorbed HPAM molecules. Concurrently, losing their stable anchoring sites, the HPAM polymer chains (brownish-yellow in color) exhibited a pronounced tendency to detach from the interface. The dynamic trajectories reveal that the displaced polymer network underwent structural relaxation and dispersed into the bulk aqueous phase, thereby disrupting the initial rigidity of the emulsion film.

**Figure 6:**
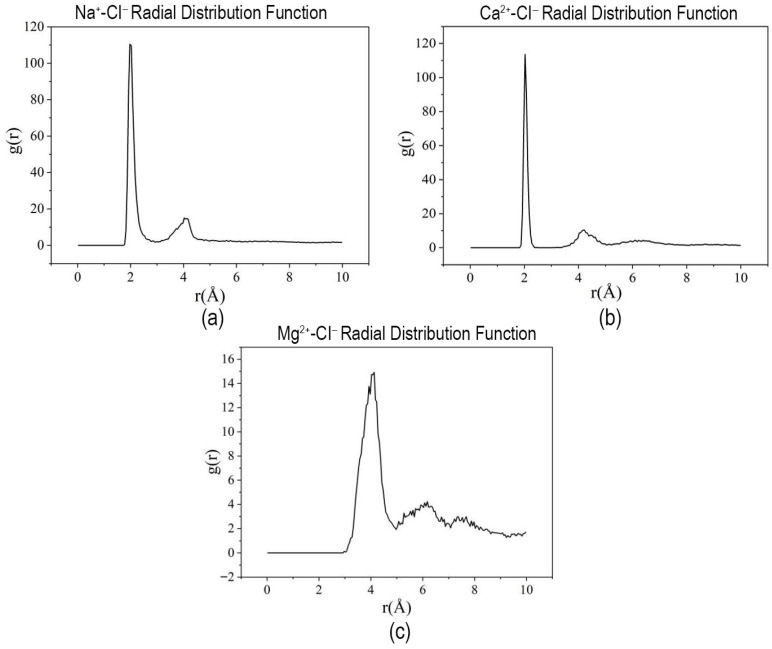
Radial distribution function (RDF) curves of HPAM and ions in Demulsifier X systems: (**a**) NaCl; (**b**) CaCl_2_; (**c**) MgCl_2_.

To further investigate the ionic coordination at the interface, the radial distribution functions (RDFs) between the oxygen atoms of the acrylate groups in HPAM and the surrounding metal cations were analyzed. As shown in Figure 6, the RDF curves exhibit maximum peaks at distances of 1.975 Å (Na^+^), 2.025 Å (Ca^2+^), and 4.125 Å (Mg^2+^). These pronounced peaks indicate strong coordination interactions between the cations and the negatively charged oxygen atoms of HPAM, which conventionally enhance the compactness and stability of the interfacial film in polymer-containing systems.

As summarized in Table 3 and Table 4, the competitive adsorption of Demulsifier X resulted in interfacial tension (IFT) values of 83.776, 77.915, and 96.461 mN/m for the NaCl, CaCl_2_, and MgCl_2_ systems, respectively. The corresponding average interfacial interaction energies (E_int_) were −4857.686, −5647.404, and −5483.660 kcal/mol.

#### 3.1.2. Interfacial Behavior of Demulsifier Y Systems

**Figure 7:**
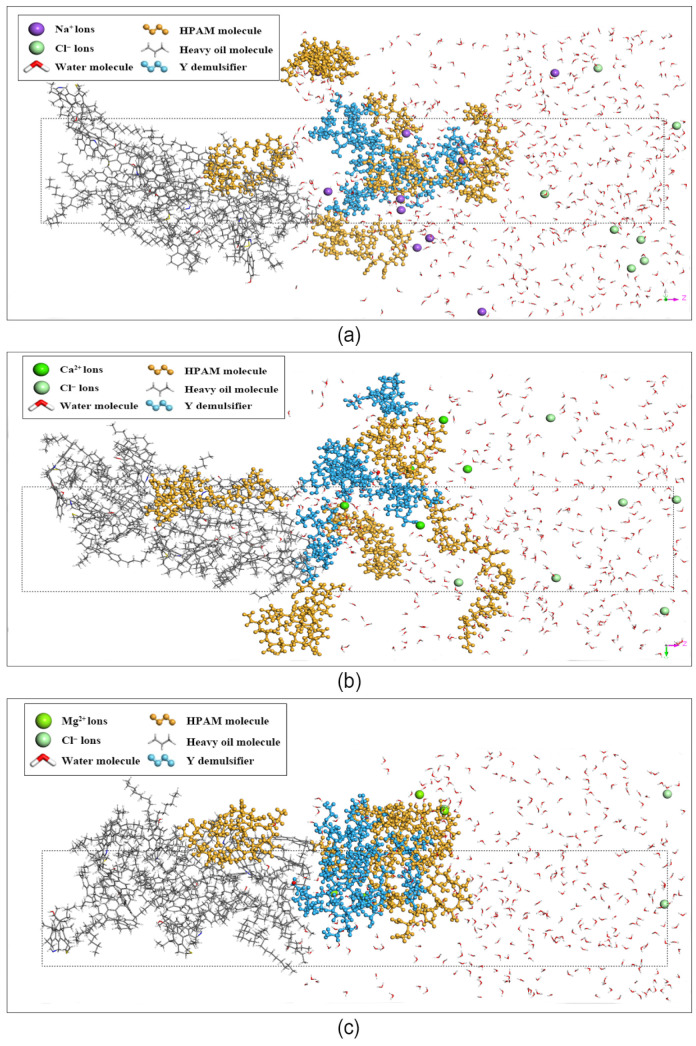
Equilibrium snapshots of the molecular dynamics simulation systems: (**a**) Y-NaCl; (**b**) Y-CaCl_2_; (**c**) Y-MgCl_2_. The molecular components are distinguished by color: Demulsifier Y (blue), HPAM chains (orange), heavy oil (grey), water (red/white), and respective ions (spheres). The dotted box indicates the boundaries of the simulation cell.

Similarly, the equilibrium snapshots for the Demulsifier Y systems in the three brine environments are depicted in Figure 7a, Figure 7b and Figure 7c, respectively. The simulation results reveal that Demulsifier Y molecules (blue chains) exhibited profound interfacial activity. Driven by their highly branched multi-arm structures, they aggressively displaced the HPAM molecules, forcing the polymer network to detach from the oil–water interface and diffuse into the bulk aqueous phase more significantly than Demulsifier X.

**Figure 8:**
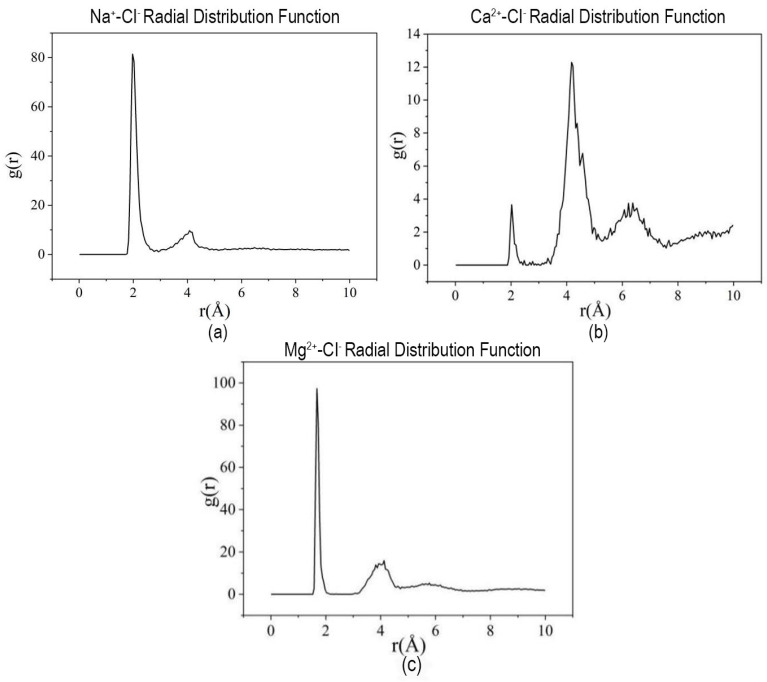
Radial distribution function (RDF) curves of HPAM and ions in Demulsifier Y systems: (**a**) NaCl; (**b**) CaCl_2_; (**c**) MgCl_2_.

The consolidated RDF curves for the Y-systems (Figure 8) display maximum peaks at 1.975 Å (Na^+^), 4.175 Å (Ca^2+^), and 1.675 Å (Mg^2+^), reflecting the altered ionic coordination environment upon the penetration of Demulsifier Y.

Quantitatively, as shown in Table 3 and Table 4, Demulsifier Y achieved higher IFT values of 86.462, 108.411, and 110.206 mN/m in the NaCl, CaCl_2_, and MgCl_2_ brines, respectively. Furthermore, it successfully reduced the absolute values of the average interfacial interaction energies to −4805.845, −4731.975, and −4319.627 kcal/mol. This significant reduction compared to Demulsifier X indicates a greater disruption of the stable oil–water–polymer interactions, highlighting the superior demulsification potential of the AP/AE series.

### 3.2. Analysis of Simulation Results

#### 3.2.1. Effect of Demulsifiers on Oil–Water Interfacial Tension

**Figure 9:**
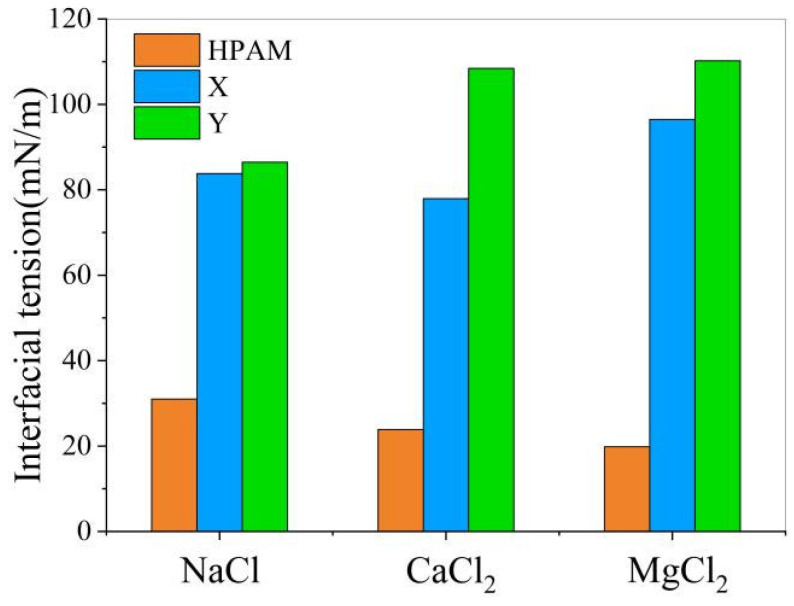
Effect of HPAM on oil–water interfacial tension.

As illustrated in Figure 9, the addition of demulsifiers to the polymer-containing oil–water system led to a distinct increase in the interfacial tension (IFT). This elevation indicates that the demulsifiers effectively disrupt the viscoelastic film, thereby reducing the stability of the oil–water interface. Specifically, when comparing the two simulated agents, Demulsifier Y exhibits a superior performance in increasing the IFT compared to Demulsifier X.

While the absolute simulated interfacial tension (IFT) values in this study (77.915–110.206 mN/m) are significantly higher than typical macroscopic measurements, this overestimation is a well-documented phenomenon in molecular dynamics (MD) simulations of complex heavy oil–water systems [28]. The deviation primarily stems from the finite-size effects of nanoscale simulation boxes (which suppress macroscopic capillary waves), the nanosecond-scale simulation duration compared to macroscopic aging times, and the parameterization constraints of the COMPASS III force field [29,30]. Furthermore, MD calculates the tension of an ideal, structurally restricted microscopic interface, unlike the dynamic, thermodynamically relaxed heterogeneous interfaces measured macroscopically. Nevertheless, theoretical models reliably capture relative trends and interaction energy variations. Consequently, comparing the relative IFT increments between Demulsifiers X and Y remains a robust method for evaluating their demulsification performance and ability to disrupt the rigid HPAM film.

Importantly, the observed IFT increase does not contradict classical emulsification theory. While low interfacial tension favors droplet formation during emulsification, breaking polymer-stabilized emulsions fundamentally requires disrupting the viscoelastic interfacial film. The partial displacement of HPAM inherently elevates the interfacial free energy, rendering the film energetically unfavorable and more prone to rupture. This thermodynamic transition weakens film elasticity and reduces the kinetic barrier for droplet coalescence. Ultimately, the observed IFT increase reflects successful interfacial destabilization rather than enhanced emulsion stability.

#### 3.2.2. Effect of Demulsifiers on Interfacial Interaction Energy

**Figure 10:**
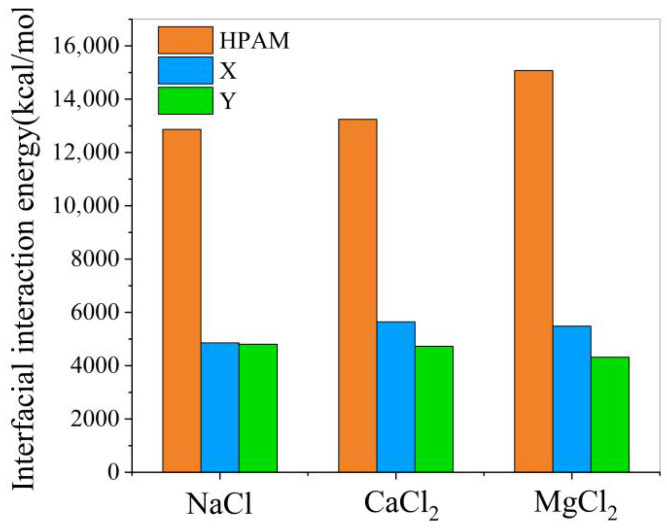
Effect of demulsifier on the interaction energy of the oil–water interface.

According to the simulation results, as illustrated in Figure 10, the introduction of demulsifiers into the polymer-containing oil–water system results in a decrease in the absolute value of the interfacial interaction energy. This reduction implies weakened interfacial interactions between the oil and aqueous phases. Furthermore, demulsifier Y demonstrates a greater capability to reduce the interfacial interaction energy compared with demulsifier X, consistent with its superior demulsification performance observed in the interfacial tension analysis.

### 3.3. Experimental Validation and Comparative Analysis

Based on the molecular dynamics (MD) simulations, Demulsifier Y exhibits a superior ability to disrupt the stable oil–water interface by more significantly increasing the interfacial tension and reducing the interfacial interaction energy. To validate these microscopic mechanistic findings, macroscopic experimental verifications were performed.

#### 3.3.1. Effect of Settling Time on Demulsification Performance

**Figure 11:**
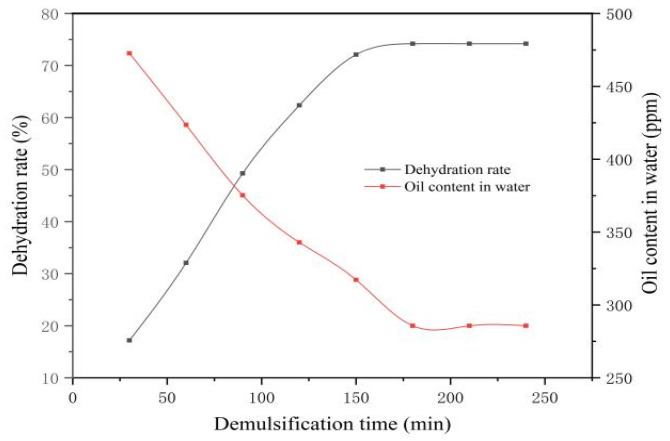
Changes in dehydration rate of X demulsifier with demulsification time. The black curve (squares) represents the dehydration rate (left axis), while the red curve (squares) indicates the residual oil content in water (right axis).

The experimental conditions were set at a temperature of 60 °C, a demulsifier concentration of 100 ppm, and a total settling time of 240 min. The macroscopic demulsification behaviors of Demulsifiers X and Y were analyzed over time. As shown in Table 5 and Figure 11, the dehydration rate for Demulsifier X exhibited a gradual upward trend from 30 to 180 min. Beyond 180 min, the dehydration rate plateaued, reaching a maximum of 74.21% with a corresponding minimum oil content in the separated water of 285.71 ppm. At this stage, the demulsifier molecules had fully diffused to the oil–water interface, resulting in a sharp phase boundary and clearly separated water. Consequently, the optimal settling time for Demulsifier X was determined to be 180 min.

**Figure 12:**
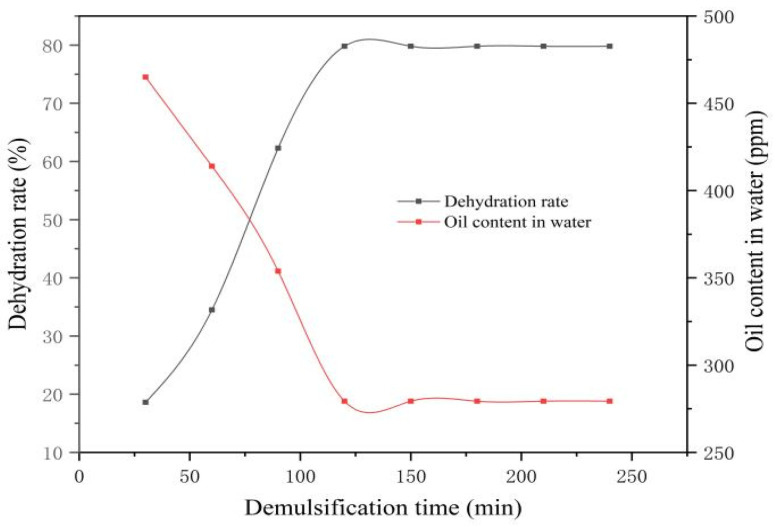
Changes in dehydration rate of Y demulsifier with demulsification time.

As indicated in Table 6 and Figure 12, the dehydration rate of the emulsion increases gradually as the demulsification time extends from 30 to 120 min. However, when the time exceeds 120 min, the change in the dehydration rate becomes insignificant, stabilizing at 79.8% with a corresponding minimum oil content in water of 279.3 ppm. At this stage, the demulsifier has fully diffused to the oil–water interface, resulting in a distinct separation boundary and clear separated water. Consequently, 120 min is determined to be the optimal demulsification time for Demulsifier Y.

In summary, under identical conditions, Demulsifier Y outperforms Demulsifier X by achieving a higher dehydration rate and lower residual oil content within a shorter optimal demulsification time.

#### 3.3.2. Effect of Temperature on Demulsification Performance

The demulsification behaviors of Demulsifiers X and Y at varying temperatures were evaluated using a fixed concentration of 120 ppm and a settling time of 150 min.

**Figure 13:**
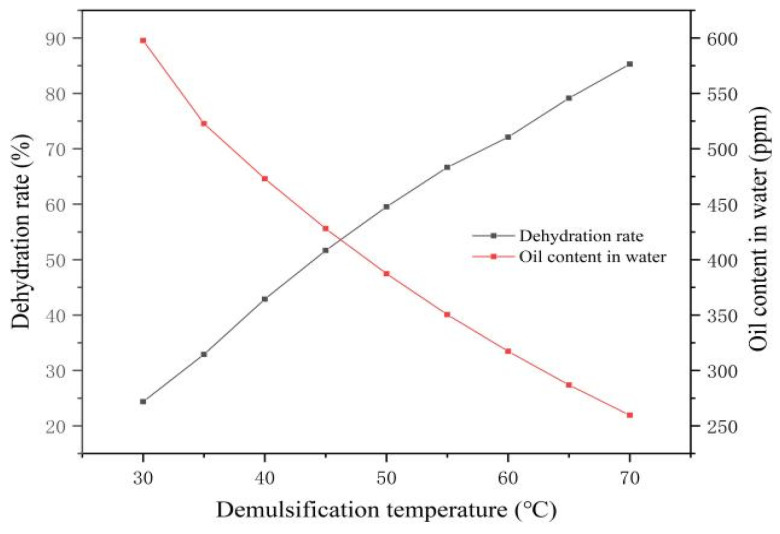
Changes in dehydration rate of X demulsifier with demulsification temperature. The relationship between demulsification temperature (30–70 °C) and the dehydration rate (black curve, left axis), as well as the residual oil content (red curve, right axis), is illustrated.

As shown in Table 7 and Figure 13, the dehydration rate increased gradually with temperature, achieving a sharp oil–water interface and clearly separated water at 70 °C. This improvement occurs because elevated temperatures kinetically accelerate the diffusion rate of demulsifier molecules from the bulk phase to the oil–water interface. Although intense thermal motion could theoretically promote desorption away from the interface, the strong chemical affinity and the significant reduction in interfacial interaction energy driven by the demulsifiers thermodynamically favor their stable adsorption, facilitating the displacement of HPAM and droplet coalescence. Consequently, it is determined that the optimal demulsification temperature for Demulsifier X under these conditions should be at least 70 °C.

**Figure 14:**
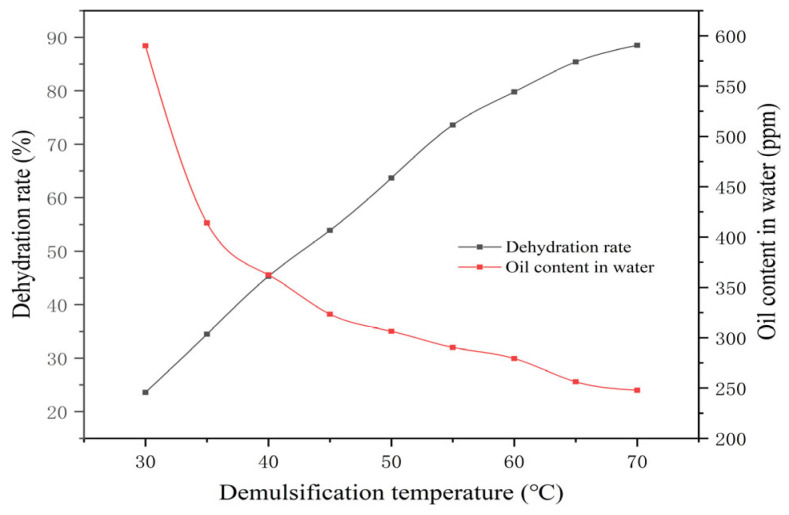
Effect of temperature on the demulsification performance.

As indicated by Table 8 and Figure 14, the dehydration rate of Demulsifier Y increased gradually with the rise in temperature. Upon reaching 60 °C, a sharp oil–water interface and clearly separated water were observed. Consequently, the operating temperature for Demulsifier Y under these conditions should be at least 60 °C.

In summary, under identical conditions of demulsifier concentration and settling time, Demulsifier Y is capable of achieving clear water separation at a lower temperature. Furthermore, when compared at the same temperature, Demulsifier Y consistently exhibits a higher dehydration rate and a lower oil content in the separated water than Demulsifier X.

#### 3.3.3. Effect of Demulsifier Concentration on Demulsification Performance

**Figure 15:**
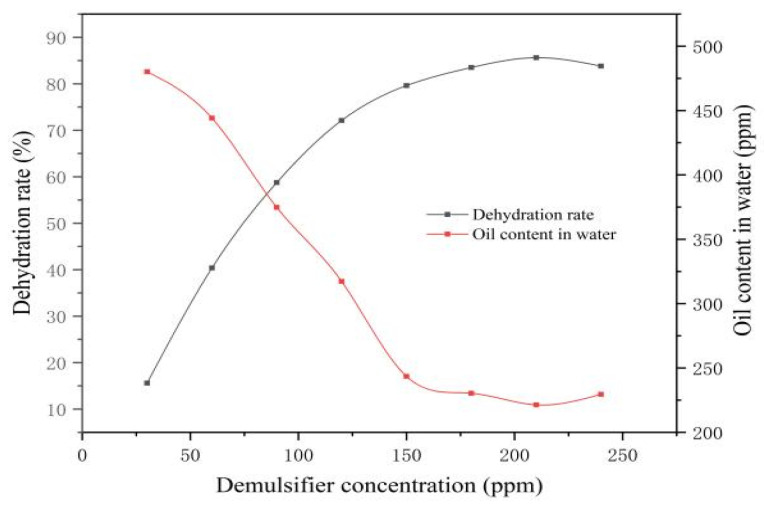
Changes in X dehydration rate with demulsifier concentration.

The demulsification behaviors of Demulsifiers X and Y at varying concentrations were evaluated at 60 °C with a 150 min settling time. As shown in Table 9 and Figure 15, the dehydration rate for Demulsifier X increased steadily from 30 to 210 ppm, achieving optimal separation with a sharp interface and clear water at 210 ppm. Beyond this dosage, the dehydration efficiency declined. Mechanistically, an optimal concentration effectively reduces interfacial shear viscosity and weakens the polymer film, thereby maximizing droplet coalescence. Conversely, a surplus of demulsifier molecules leads to the formation of a new, stable interfacial film, which sterically hinders coalescence and reduces overall efficiency. Thus, 210 ppm was determined as the optimal concentration for Demulsifier X.

**Figure 16:**
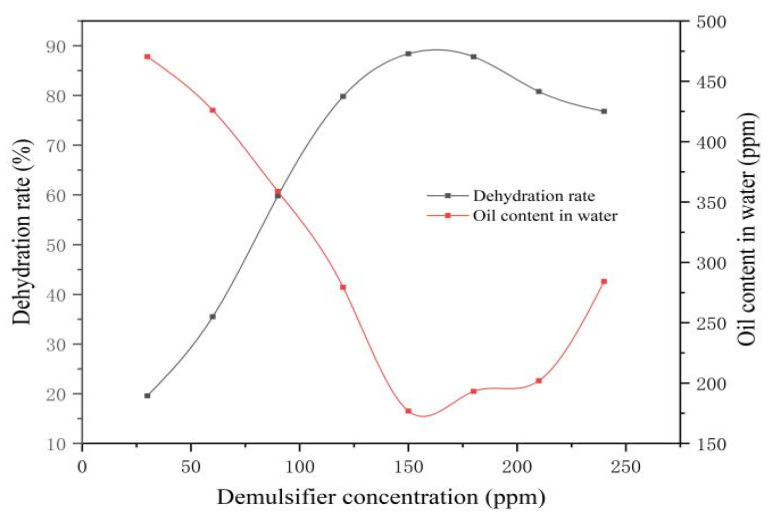
Changes in Y dehydration rate with demulsifier concentration.

As illustrated in Table 10 and Figure 16, the dehydration rate of the emulsion significantly improved as the concentration of Demulsifier Y increased within the range of 30 to 150 ppm. However, beyond this range, further increasing the concentration resulted in a decreasing trend in the dehydration rate. This phenomenon indicates that the demulsifier concentration has a critical impact on dehydration efficiency, and an optimal concentration exists. Under constant temperature and settling time conditions, the dehydration rate peaked at 150 ppm. At this dosage, a sharp oil–water interface and clearly separated water were observed. Consequently, 150 ppm was identified as the optimal concentration for Demulsifier Y.

In summary, under identical conditions of temperature and settling time, the optimal concentration of Demulsifier Y is lower than that of Demulsifier X. Furthermore, at their respective optimal concentrations, Demulsifier Y exhibits superior demulsification performance. The higher IFT increase and stronger reduction in interfacial interaction energy induced by Demulsifier Y are consistent with its superior dehydration efficiency observed experimentally, establishing a clear structure–mechanism–performance relationship.

## 4. Conclusions

Based on MD simulations, this study clarifies that the fundamental mechanism of demulsification is governed by competitive adsorption and interfacial displacement. The demulsifier molecules effectively diffuse onto the oil–water interface, where they displace and detach the polymer molecules that were originally and stably adsorbed. This process alters the physicochemical properties of the interfacial film, thereby destabilizing the emulsion structure and facilitating the oil–water separation process.

Through a comprehensive analysis combining microscopic simulations and macroscopic bottle tests, Demulsifier Y (AP/AE series polyamine polyethers) demonstrates significantly superior overall demulsification performance compared to Demulsifier X (SP/BP series alcohol polyethers). Microscopic simulations reveal that Demulsifier Y more effectively increases the oil–water interfacial tension and reduces the interfacial interaction energy. Furthermore, macroscopic experiments confirm that, under identical conditions, Demulsifier Y achieves a higher dehydration rate (peaking at 88.4%) and a lower residual oil content in the separated water.

Demulsifier Y exhibits superior comprehensive benefits in practical applications. It requires a shorter optimal demulsification time (120 min vs. 180 min for Type X), a lower effective temperature (60 °C vs. 70 °C), and a lower optimal concentration (150 ppm vs. 210 ppm). These findings indicate that Demulsifier Y can achieve high-efficiency oil–water separation with reduced energy consumption and chemical dosage, making it a more cost-effective solution.

## Figures and Tables

**Table 1:** Key parameters and structural views of established molecular models.

Model Category	Representative Component	Construction Details	Force Field	Validation and Equilibration	Structural View
Heavy Oil Molecular Model	SARA Components: Saturates, Aromatics, Resins, Asphaltenes	The initial density was set to 1.0 g/cm^3^. The simulation cell with periodic boundary conditions was constructed using the Amorphous Cell module.	COMPASS III	Equilibrium density: 0.958 g/cm^3^; relative error: 1.46% compared to experimental value (0.944 g/cm^3^).	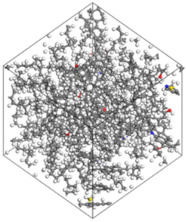
Ion Model	Na^+^, Ca^2+^, Mg^2+^, Cl^−^	Constructed using the intrinsic ion force field parameters and libraries within Materials Studio.	COMPASS III	Charge neutrality achieved; directly employed for the construction of the aqueous phase.	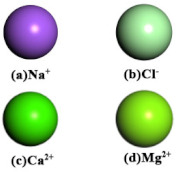
Polymer Model	Partially Hydrolyzed Polyacrylamide (HPAM) (DP = 20, DH = 25%)	Atactic HPAM chain constructed; Geometry Optimization and Annealing performed via the Forcite module to obtain the lowest-energy stable conformation.	COMPASS III Forcite	Structural stability achieved following geometry optimization and annealing cycles.	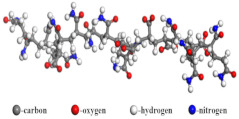
Saline Water Model	H_2_O + NaCl/CaCl_2_/MgCl_2_	A pure water box (1000 molecules) was first constructed and optimized; ions were added based on actual composition and cation ratios (Cl^−^ count fixed at 10).	Amorphous Cell COMPASS III Forcite	System energy convergence; uniform distribution of ions within the solvent.	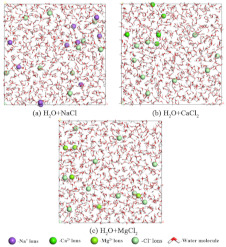

**Table 2:** Typical physicochemical properties of Demulsifier X and Demulsifier Y.

Properties	Demulsifier X (SP/BP Series)	Demulsifier Y (AP/AE Series)
Initiator Type	Fatty alcohol	Polyamine
Average Molecular Weight	~2500 g/mol	~4000 g/mol
HLB Value	6.5–8.0	7.5–9.5
Solubility	Oil-soluble/Water-dispersible	Water-soluble/Oil-dispersible
Macroscopic Interfacial Tension	~15–20 mN/m	~10–15 mN/m

**Note:** The typical physicochemical properties of the SP/BP and AP/AE series demulsifiers presented in Table 2 were derived from representative chemical profiles summarized in the textbook *Oilfield Chemicals* by Luo [24] and commonly reported industrial data. These parameters serve as the macroscopic reference for the molecular models constructed in this study, where the molecular weight and HLB values correspond to the 1:1 molar ratio of EO to PO units adopted in the simulations.

**Table 3:** Interfacial tension of different emulsion systems.

Demulsifier	Brine System	Pxx (GPa)	Pyy (GPa)	Pzz (GPa)	Lz (Å)	Interfacial Tension (mN/m)
X	NaCl	−0.046	−0.043	−0.039	151.827	83.776
X	CaCl_2_	−0.055	−0.055	−0.050	151.503	77.915
X	MgCl_2_	−0.065	−0.067	−0.060	151.215	96.461
Y	NaCl	−0.056	−0.056	−0.051	157.203	86.462
Y	CaCl_2_	−0.055	−0.052	−0.047	156.879	108.411
Y	MgCl_2_	−0.054	−0.055	−0.047	156.591	110.206

**Table 4:** Interfacial interaction energy of different emulsion systems.

Demulsifier	Brine System	EA-B-C-D (kcal/mol)	EA (kcal/mol)	EB (kcal/mol)	EC (kcal/mol)	ED (kcal/mol)	Eint (kcal/mol)
X	NaCl	−13,255.458	165.867	−1513.468	476.591	−7526.763	−4857.686
X	CaCl_2_	−14,103.432	173.618	−1053.733	450.508	−8026.420	−5647.404
X	MgCl_2_	−14,453.413	201.102	−1170.564	448.248	−8448.539	−5483.660
Y	NaCl	−13,286.991	207.311	−1311.032	214.616	−7592.042	−4805.845
Y	CaCl_2_	−14,188.801	237.810	−1269.822	207.064	−8631.878	−4731.975
Y	MgCl_2_	−14,518.886	198.402	−1501.379	243.130	−9139.412	−4319.627

**Table 5:** Demulsification status of X demulsifier under different demulsification times.

Settling Time (min)	Dehydration Rate (%)	Oil Content in Water (ppm)	Interfacial Status	Quality of Separated Water
30	17.19	472.73	Ragged	Turbid; Oil Film on Wall
60	32.09	423.59	Ragged	Turbid; Oil Film on Wall
90	49.28	375.25	Ragged	Slightly Turbid
120	62.36	342.98	Moderately Sharp	Clear
150	72.1	317.24	Moderately Sharp	Clear
180	74.21	285.71	Sharp	Clear
210	74.21	285.71	Sharp	Clear
240	74.21	285.71	Sharp	Clear

**Table 6:** Demulsification status of Y demulsifier under different demulsification times.

Settling Time (min)	Dehydration Rate (%)	Oil Content in Water (ppm)	Interfacial Status	Quality of Separated Water
30	18.6	465	Ragged	Turbid; Oil Film on Wall
60	34.5	414	Ragged	Slightly Turbid
90	62.3	353.8	Moderately Sharp	Slightly Turbid
120	79.8	279.3	Sharp	Clear
150	79.8	279.3	Sharp	Clear
180	79.8	279.3	Sharp	Clear
210	79.8	279.3	Sharp	Clear
240	79.8	279.3	Sharp	Clear

**Table 7:** Demulsification status of X demulsifier at different demulsification temperatures.

Settling Time (min)	Dehydration Rate (%)	Oil Content in Water (ppm)	Interfacial Status	Quality of Separated Water
30	24.37	597.69	Ragged	Turbid; Oil Film on Wall
35	32.89	522.72	Ragged	Turbid; Oil Film on Wall
40	42.86	472.98	Ragged	Turbid; Oil Film on Wall
45	51.66	427.97	Ragged	Turbid; Oil Film on Wall
50	59.53	387.24	Ragged	Turbid; Oil Film on Wall
55	66.65	350.39	Moderately Sharp	Slightly Turbid
60	72.1	317.24	Moderately Sharp	Slightly Turbid
65	79.14	286.87	Moderately Sharp	Clear
70	85.3	259.57	Sharp	Clear

**Table 8:** Demulsification status of Y demulsifier at different demulsification temperatures.

Settling Time (min)	Dehydration Rate (%)	Oil Content in Water (ppm)	Interfacial Status	Quality of Separated Water
30	23.6	590	Ragged	Turbid; Oil Film on Wall
35	34.5	414	Ragged	Turbid; Oil Film on Wall
40	45.3	362.4	Ragged	Turbid; Oil Film on Wall
45	53.9	323.4	Ragged	Turbid; Oil Film on Wall
50	63.7	306.5	Ragged	Slightly Turbid
55	73.6	290.4	Moderately Sharp	Slightly Turbid
60	79.8	279.3	Sharp	Clear
65	85.4	256.2	Sharp	Clear
70	88.5	247.8	Sharp	Clear

**Table 9:** Demulsification status of X demulsifier at different demulsifier concentrations.

Settling Time (min)	Dehydration Rate (%)	Oil Content in Water (ppm)	Interfacial Status	Quality of Separated Water
30	15.589	480.14	Ragged	Turbid; Oil Film on Wall
60	40.38	444.18	Ragged	Turbid; Oil Film on Wall
90	58.74	374.76	Ragged	Turbid; Oil Film on Wall
120	72.1	317.24	Ragged	Slightly Turbid
150	79.6	243.42	Ragged	Slightly Turbid
180	83.5	230.36	Moderately Sharp	Slightly Turbid
210	85.6	221.28	Sharp	Clear
240	83.8	229.53	Sharp	Clear

**Table 10:** Demulsification status of Y Demulsifier at different Demulsifier concentrations.

Settling Time (min)	Dehydration Rate (%)	Oil Content in Water (ppm)	Interfacial Status	Quality of Separated Water
30	19.6	470.4	Ragged	Turbid; Oil Film on Wall
60	35.5	426	Ragged	Slightly Turbid
90	59.8	358.8	Ragged	Slightly Turbid
120	79.8	279.3	Sharp	Clear
150	88.4	176.8	Sharp	Clear
180	87.8	193.16	Sharp	Clear
210	80.8	202	Sharp	Clear
240	76.8	284.16	Sharp	Clear

## Data Availability

The original contributions presented in this study are included in the article. Further inquiries can be directed to the corresponding authors.
